# Controlling Mesenchyme Tissue Remodeling via Spatial Arrangement of Mechanical Constraints

**DOI:** 10.3389/fbioe.2022.833595

**Published:** 2022-02-18

**Authors:** Tackla S. Winston, Chao Chen, Kantaphon Suddhapas, Bearett A. Tarris, Saif Elattar, Shiyang Sun, Teng Zhang, Zhen Ma

**Affiliations:** ^1^ Department of Biomedical and Chemical Engineering, Syracuse University, Syracuse, NY, United States; ^2^ BioInspired Syracuse Institute for Materials and Living Systems, Syracuse University, Syracuse, NY, United States; ^3^ Department of Mechanical and Aerospace Engineering, Syracuse University, Syracuse, NY, United States; ^4^ Department of Chemical and Petroleum Engineering, University of Kansas, Lawrence, KS, United States

**Keywords:** tissue remodeling, tissue mechanics, human induced pluripotent stem cell (hiPSC), finite element analyses, mesenchymal stem cells, tissue morphogenesis

## Abstract

Tissue morphogenetic remodeling plays an important role in tissue repair and homeostasis and is often governed by mechanical stresses. In this study, we integrated an *in vitro* mesenchymal tissue experimental model with a volumetric contraction-based computational model to investigate how geometrical designs of tissue mechanical constraints affect the tissue remodeling processes. Both experimental data and simulation results verified that the standing posts resisted the bulk contraction of the tissues, leading to tissue thinning around the posts as gap extension and inward remodeling at the edges as tissue compaction. We changed the geometrical designs for the engineered mesenchymal tissues with different shapes of posts arrangements (triangle vs. square), different side lengths (6 mm vs. 8 mm), and insertion of a center post. Both experimental data and simulation results showed similar trends of tissue morphological changes of significant increase of gap extension and deflection compaction with larger tissues. Additionally, insertion of center post changed the mechanical stress distribution within the tissues and stabilized the tissue remodeling. This experimental-computational integrated model can be considered as a promising initiative for future mechanistic understanding of the relationship between mechanical design and tissue remodeling, which could possibly provide design rationale for tissue stability and manufacturing.

## Introduction

Tissue remodeling is a biological process of reorganization of living tissues under physiological or pathological conditions. For example, during prenatal and postnatal developments, the remodeling of vascular networks through angiogenesis and vascular regression plays an important role in maintaining and optimizing nutrient and oxygen distribution (Gibbons and Dzau, 1994; Rodriguez-Porcel et al., 2006; Rouwkema and Khademhosseini, 2016). During wound healing, fibrous mesenchyme tissue undergoes a rapid remodeling process to restore tissue integrity and function (Hinz et al., 2012; Nour et al., 2019; Smith et al., 2019). It has been indicated that tissue remodeling processes in normal development or disease progression strongly depend on the biomechanical properties of bulk tissues and associated mechanobiological responses of individual cells (Legant et al., 2012; Sakar et al., 2016; Bose et al., 2019; Malandrino et al., 2019). Understanding how the mechanical constraints can manipulate tissue remodeling would potentially provide us mechanistic insights into the structure-function relationship of biological tissues.

By integrating biological tissues with engineered materials and designed structures, numerous efforts have been devoted to elucidating the relationship between tissue remodeling and fundamental biophysics. Three-dimensional (3D) *in vitro* tissue model systems have gained more interests from the field of biomechanics and tissue engineering, since they allow for the assessment of mechanical properties, force generation, and dynamic remodeling of biological tissue constructs. Particularly, microfabricated platforms with standing posts have been extensively used to generate biological tissues for investigating the relationship between tissue morphology, cellular behaviors, matrix elasticity, cantilever mechanics, and geometric constraints (Legant et al., 2009; Zhao et al., 2013; Ramade et al., 2014). It has been shown that the variation of post design would induce morphological instability of the contractile tissues (Christensen et al., 2020). An experimental-computational integrated model was developed based on the morphological evolution of these mechanically constrained tissues. This model can predict the necking and failure of the biological tissues over time and provide rational design of functional tissues with reduced local stresses (Wang et al., 2013). In previous study, microvascular meshes were fabricated using a micropillar-based platform. In this system, the inner micropillars served as geometric templates to guide the self-assembly of endothelial cells with a controlled order, while the boundary micropillars served as the anchoring points to prevent the tissue contraction and increase the stability of these microvascular meshes. Correspondingly, a computational model was established to describe the tissue assembly by incorporating both passive elastic contribution from the fibrin matrix and an active contractile contribution from the cells (Song et al., 2019). More recently, active mechanical actuation was introduced to these engineered platforms to perform the tensile testing for the engineered tissues, to assess their stress relaxation and recovery responses (Liu et al., 2016; Walker et al., 2020). Mechanical responses of tissues to the active actuations were used to create a Hill-type simulation model that coupled actomyosin dynamics with viscoelastic properties.

Mechanical modeling of contraction behaviors of tissue materials is an active research topic in the biomechanics field (Rajagopal et al., 2018; Dubois et al., 2019; Dey et al., 2020). A small-strain biochemo-mechanical model was created to simulate the cell contractile behaviors and stress fiber formation (Deshpande et al., 2006; Pathak et al., 2008). In this model, directional stress fibers were developed in response to the stimulus signals, which led to predictable cell contractility following the classical Hill’s equation. The timescale of such remodeling response was related to the formation of stress fibers within the biological cells. This model was further developed into a 3D large-strain model to predict the necking instability of engineered microtissues supported by two standing posts (Wang et al., 2013). In addition to these stress fiber models, a computational model based on volumetric contraction and surface stress contraction was developed to study the equilibrium 3D deformation and reconstruction of contractile microtissues (Mailand et al., 2019; Kim et al., 2020). In general, the tissue morphology of constrained microtissues was analyzed within 72 h (3 days), while the early work of tissue morphological evolution examined the tissue failure over 7 days (Wang et al., 2013), which is the longest study that we are aware of. For the tissue sizes, the largest tissues used for biomechanical analysis had a designed 2.5 × 2.5 mm^2^ square geometry (Dubois et al., 2019), which were still significantly smaller than the tissues that we created. Hence, these models focused on microtissues in the order of hundreds of microns, while proper modeling approach to study the morphological remodeling of large tissue constructs over a long period of time is still underexplored.

Despite these previous efforts, it is still elusive how tissue remodeling process evolves with different spatial arrangements of the standing posts, which could induce different patterns of mechanical constraints to these engineered tissue constructs. To address this, we created an *in vitro* tissue model from the mesenchymal stromal cells derived from human induced pluripotent stem cells (hiPSC-MSCs) using fabricated devices with standing posts. This experimental model system allowed us to determine how different spatial arrangements of standing posts would influence the progression of tissue remodeling. Furthermore, we integrated a computational model based on volumetric contraction to predict the deformation across the constrained tissues. Comparing to previous works, we identified a unitless parameter of the ratio between contraction modulus and elastic modulus that can be used to determine the tissue remodeling process due to the volume constraints by the geometrical designs. By tightly coupling tissue fabrication and multiscale modeling, this experimental-computational integrated model would shed light on tissue mechanics and morphological evolution under different designs of mechanical constraints.

## Results and Discussion

The PDMS devices with different spatial arrangements of standing posts were generated from 3D-printed ABS molds and coated with 10% Pluronic Acid. To create a tissue in a PDMS-based multi-post device, hiPSC-MSCs were suspended in the collagen type I solution and seeded into the device (Supplementary Figure S1A). Within 24 h, the cells self-assembled into a dense 3D tissue construct around the standing posts and formed into large tissue constructs. Supplementary Figure S1B demonstrates a typical geometric design to create triangular tissues. We were able to fix the tissues in the devices for fluorescent microscopy imaging and scanning electron microscopy (SEM) imaging. We observed that cell nuclei were evenly distributed within the tissue construct, and actin stress fibers were aligned in the direction of tension development that was oriented based on the post arrangement (Supplementary Figures S1C,D). The formation of dense stress fibers was found at the edges of the tissue construct, especially where the tissue was anchored by the standing posts (Supplementary Figures S1E,F). In this study, we primarily focused on two geometric designs of the tissue constructs: triangular shape with three standing posts and square shape with four standing posts. For each geometric design, we changed the distance between the posts (side length = 6 mm or 8 mm). The standing PDMS posts (diameter of 1 mm and height of 4 mm) were stiff enough to avoid significant deformation during the remodeling process. Moreover, we imposed a center post for each design to study how it would affect the distribution of mechanical constraints and the progression of tissue remodeling.

**FIGURE 1:**
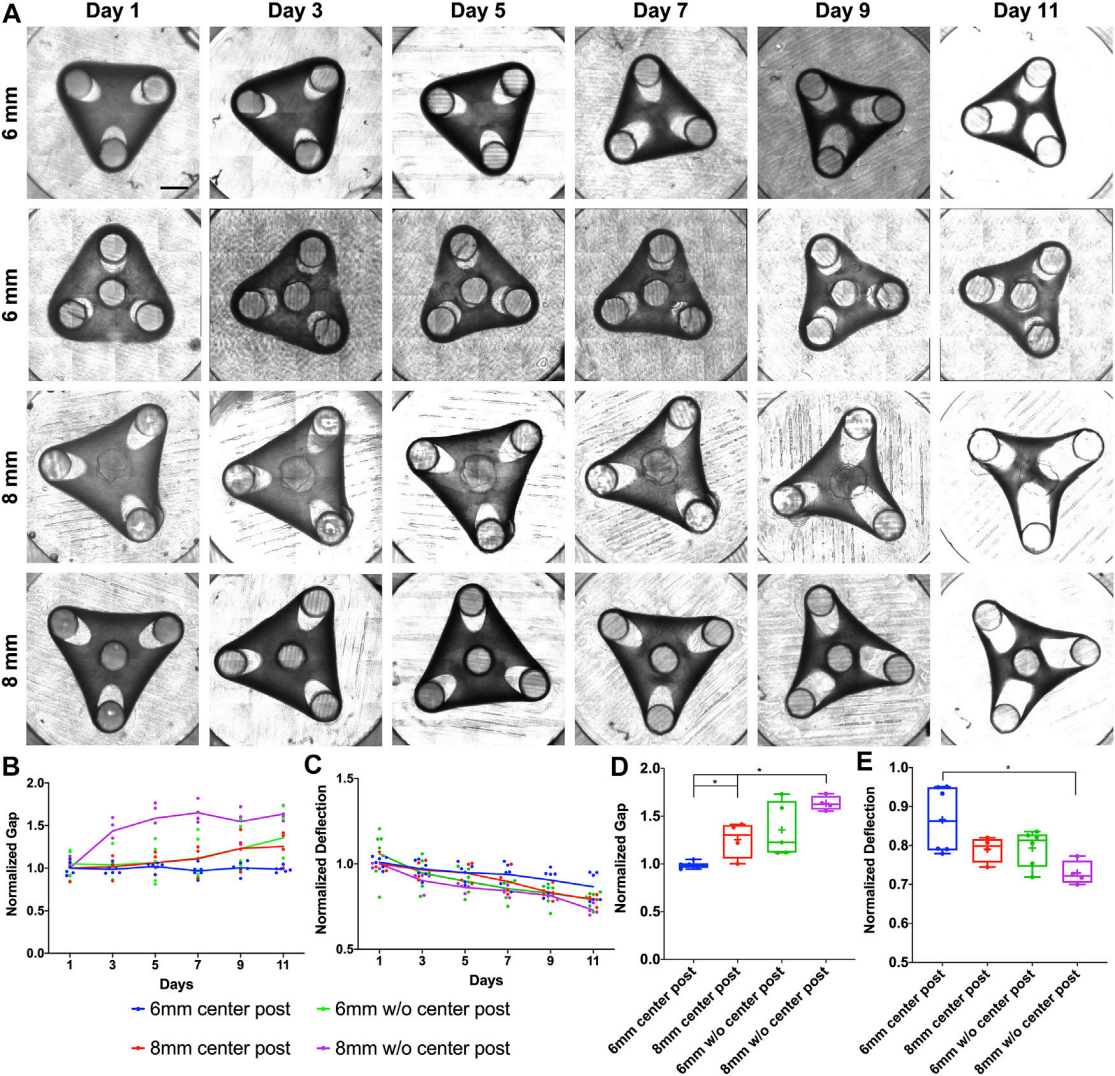
Experimental results of morphological evolution of triangular mesenchymal tissues. **(A)** Representative images of tissue remodeling over 11 days for the triangular tissues of different side lengths and with or without center post. **(B)** Tissue thinning around the post led to gap extension, and **(C)** inward remodeling at the edge led to deflection compaction. The gap and deflection were measured every 2 days for tissue comparison (▲6wC, ▲8wC, ▲6woC and ▲8woC). Taking the measurements on Day 11, **(D)** the gap was larger and **(E)** the deflection was smaller for the triangular tissues with longer side length and without the center post. Scale bar: 1 mm *p < 0.05.

To characterize the tissue remodeling, two parameters of gap and deflection were measured every 2 days. The gap is defined as the distance between the furthest tip of the expanded loop to the post center. In our work, the deflection is defined as the distance between the tissue’s outer edge and the geometric center of designed triangles or squares, which is opposite to the common definition of “*deflection*” referring to the displacement of tissue’s outer edge from its original position, since defining and measuring the original position induced significant variations in the analysis (Supplementary Figure S2A). First, we examined the tissue remodeling from Day 1 to Day 11 for the triangular tissue designs with different post distances and insertion of center post (Figure 1A). We observed that the tissues of 8 mm side length without a center post (▲8woC) had significant gap elongation as early as Day 3, while tissues of 8 mm with center post (▲8wC) only showed an increase of gap starting Day 7 (Figure 1B). For 6 mm side length, the tissues without a center post (▲6woC) showed an increase of gap starting Day 7, while the gap for the tissues with a center post remained relatively consistent over 11 days. Tissue compaction induced a decrease of deflection for all the tissues, but the tissues without center posts (▲6woC and ▲8woC) showed a faster decreasing trend, comparing to their counterparts with center posts (▲6wC and ▲8wC), respectively (Figure 1C). Taking the measurements of gap and deflection at Day 11, we observed that the increase of side length would significantly induce the gap extension and deflection compaction (Figures 1D,E). More importantly, the insertion of center posts reduces the gap extension, thus improves the tissue stability. To assess how the size of center posts would influence the tissue remodeling process, we reduced the diameter of the center posts from 2 to 1 mm for the 8 mm triangular tissues (▲8wC-R, Supplementary Figure S3A). The measurements of gap and defection of ▲8wC-R tissues were comparable to the ▲8wC tissues, though the post size was much smaller (Supplementary Figures S3B,C). Measurements at Day 11 showed that the tissues with center posts (▲8wC and ▲8wC-R) had significantly smaller gaps than the ones without a center post (▲8woC) (Figure 3D), but no significant difference on the deflection (Supplementary Figure S3E).

To simulate the morphogenetic evolution of tissue remodeling, we used a bulk contraction model (Kim et al., 2020) with a time-dependent free-energy function to predict the minimal principal logarithmic strain distribution and the corresponding tissue deformation represented by gap and deflection. To model the maximal tissue contractility with fully developed stress fibers, the bulk contraction modulus of the tissue is fixed, while the elastic moduli are relaxed due to the viscoelastic behaviors of biological tissues. For a tissue material point located at the coordinates (*X*_1_, *X*_2_, *X*_3_) before deformation, the coordinates after deformation are (*x*_1_, *x*_2_, *x*_3_). The deformation gradient of the material is defined as *F*_*iK*_ = ∂*x*_*i*_/∂*X*_*K*_ *.* The first invariant of the right Cauchy-Green deformation tensor *I* = *F*_*iK*_ *F*_*iK*_. *J* is the determinant of *F*_*iK*_ which represents the volume ratio before and after deformation. The free energy of tissue is

$$W=\frac{\mu }{2}\left(\bar{I}-3\right)+\frac{\kappa }{2}{\left(\ln J\right)}^{2}+\eta J,$$ (1)

where $\bar{I}=IJ^{-2/3}$ is the first deviatoric strain invariant. Here the first two terms denote the energy associated with passive material component modeled with a compressible neo-Hookean model, where *μ* and *κ* are the shear and bulk moduli. The third term denotes the energy associated with the active volumetric bulk contraction where *η* is the contraction modulus (Kim et al., 2020). The corresponding stress–strain relation is:

$$\sigma_{ij}=\frac{\mu }{J}\left(J^{-2/3}F_{iK}F_{jK}-\frac{1}{3}J^{-2/3}F_{mK}F_{mK}\delta_{ij}\right)+\left(\kappa \frac{\ln J}{J}+\eta \right)\delta_{ij}.$$ (2)

To model the evolution of the tissue remodeling, we presume the shear and bulk moduli decay exponentially with time *t*:

$$\mu =\mu_{0}\exp\left(1-\frac{t}{\tau }\right)\;and\;\kappa =\kappa_{0}\exp\left(1-\frac{t}{\tau }\right).$$ (3)

The contraction modulus *η* is fixed as a constant. In our modeling, we fix the parameters as following (Mailand et al., 2019): the contraction modulus *η* = 11 kPa, the elastic modulus *E*_0_ = 18.33 kPa, and the Poisson’s ratio *υ* = 0.3. The initial shear and bulk moduli can be calculated through: *μ*_0_ = *E*_0_/(2 (1+*υ*)), *κ*_0_ = *E*_0_/(3 (1–2*υ*)). The characteristic time *τ* is 4 days.

**FIGURE 2:**
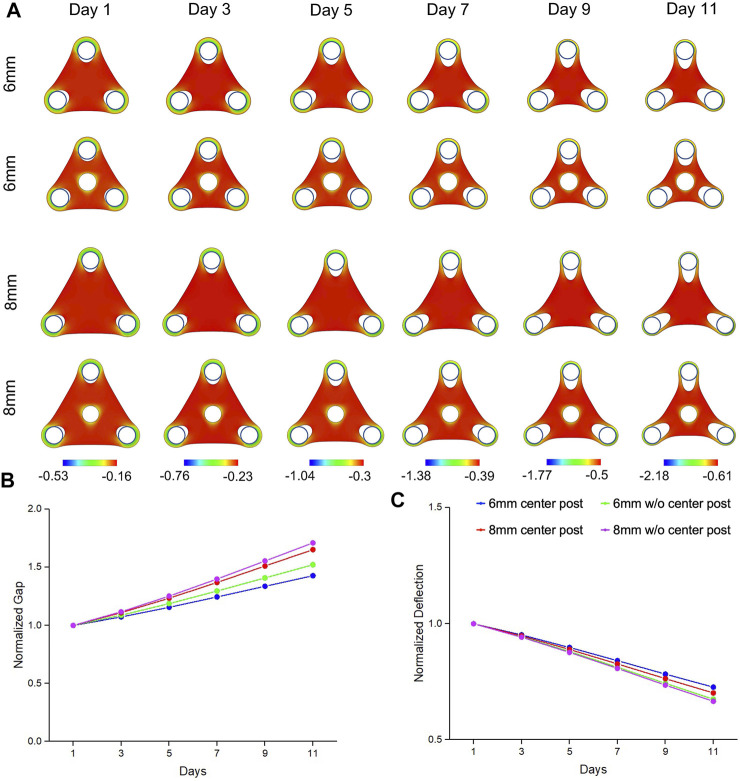
Simulation results of morphogenic evolution of triangular mesenchymal tissues. **(A)** A volumetric contraction model with a time-dependent free-energy function was used to simulate the morphological evolution of the triangular tissues. The color represents the minimal principal logarithmic strain distribution. The computational model was able to replicate the trends in morphological changes of gap extension and deflection compaction that were observed from the experimental model. For the tissue comparison (▲6wC, ▲8wC, ▲6woC and ▲8woC), **(B)** the gap was larger and **(C)** the deflection was smaller for the triangular tissues with longer side length and without the center post.

Our simulations were able to reproduce the morphogenetic evolution of the tissue remodeling, including tissue thinning around the standing posts that led to the gap extension and tissue compaction at the edge that led to the deflection (Figure 2A). We observed that mechanical strains were concentrated around the standing posts, including the center post, which suggested that insertion of center post changed the internal mechanical stress distribution of the tissue and altered the tissue remodeling. We believe that the bulk contraction resulted in a reduction of tissue surface area and smoothing of features with high curvatures. The geometrical constraints from standing posts resisted the bulk contraction and created the concave shape at the edge of the tissues. Our simulation results also showed that gap extension from the triangular tissues of 8 mm was significantly larger than the one of the tissues of 6 mm, and more importantly, the insertion of center post reduced the gap extension (Figure 2B). Accordingly, deflection compaction was more obvious from larger tissues without center posts (Figure 2C). The trend of gap extension and deflection compaction from the simulation results was consistent with the experimental results observed from the tissues with different designs of geometrical constraints. Additionally, we also simulated the morphogenetic evolution of the tissues with smaller center post (Supplementary Figure S4A). A similar trend was observed that reduction of gap extension and tissue compaction with an increase of post size, but the difference among different center post designs was much smaller than the experimental results (Supplementary Figures S4B,C).

**FIGURE 3:**
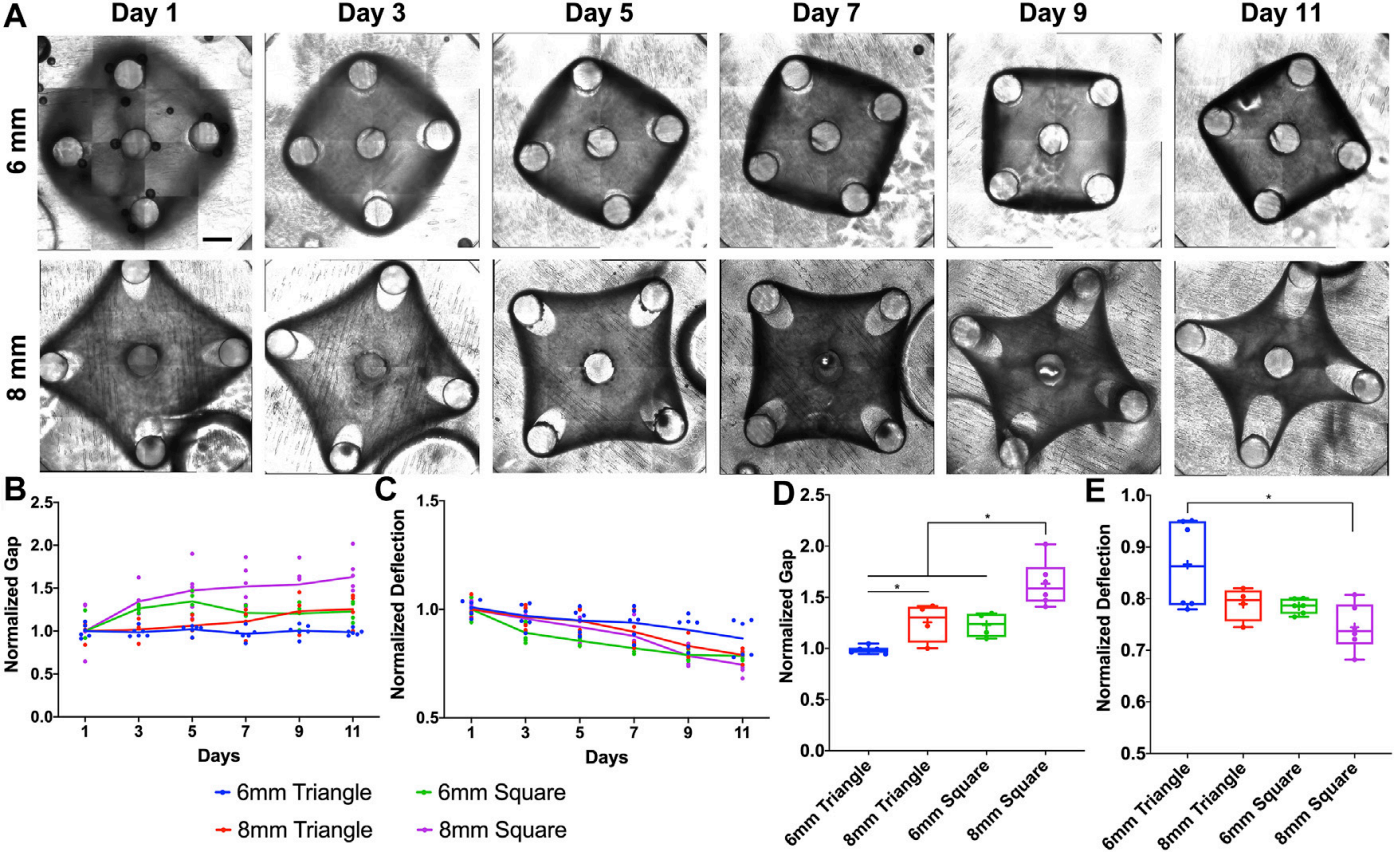
Experimental results of morphological evolution of square mesenchymal tissues. **(A)** Representative images of tissue remodeling over 11 days for the square tissues of different side lengths. **(B)** Tissue thinning around the post led to gap extension, and **(C)** inward remodeling at the edge led to deflection compaction. The gap and deflection were measured every 2 days for tissue comparison (▲6wC, ▲8wC, ∎6wC and ∎8wC). Taking the measurements on Day 11, **(D)** the gap was larger and **(E)** the deflection was smaller for the square tissues with longer side length. Scale bar: 1 mm *p < 0.05.

To further investigate how the tissue geometry would affect the tissue remodeling process, we created square tissues with different side lengths (6 and 8 mm). We were not able to maintain a square tissue without a center post for 11 days, since the tissue thinning at the post regions with dramatic gap extension caused the tissue failure. To improve the stability of tissue morphological remodeling, we imposed the center post for all the square tissues (Figure 3A). For the gap measurement, the square tissues (∎6wC and ∎8wC) showed significant gap extension as early as Day 3, while ▲8wC tissues only started at Day 7 and ▲6wC kept relatively consistent throughout 11 days (Figure 3B). Similarly, the deflection of squared tissues also showed a faster decreasing trend than the deflection of triangular tissues (Figure 3C). Taking the measurements at Day 11, we observed a significantly larger gap and more deflection from ∎8wC tissues, indicating the morphological instability during the tissue remodeling (Figures 3D,E).

**FIGURE 4:**
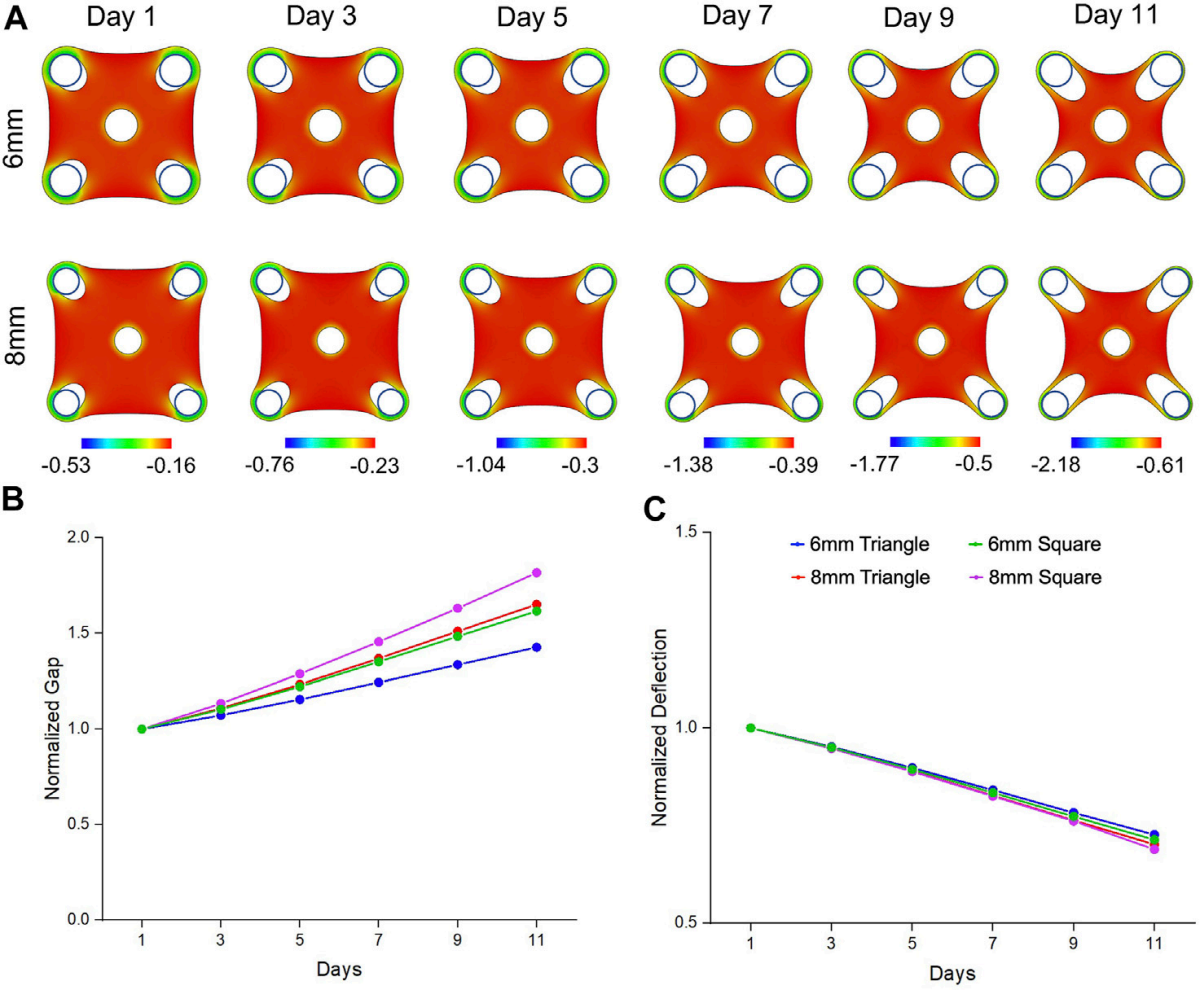
Simulation results of morphogenic evolution of square mesenchymal tissues. **(A)** A volumetric contraction model with a time-dependent free-energy function was used to simulate the morphological evolution of the square tissues. The color represents the minimal principal logarithmic strain distribution in the tissue. The computational model was able to replicate the trends in morphological changes of gap extension and deflection compaction that were observed from the experimental model. For the tissue comparison (▲6wC*,* ▲8wC*,* ∎6wC and ∎8wC), **(B)** the gap was larger and **(C)** the deflection was smaller for the square tissues with longer side length.

Similarly, from our simulation results, the square tissues showed concave shape and remodeled inwards due to the bulk contraction (Figure 4A). With the same trend of experimental results, simulation data also showed that ∎8wC tissues had faster and larger gap extension than the ∎6wC tissues (Figure 4B). Meanwhile, the square tissues had faster and larger gap extension than the triangular tissues with the same side length (∎8wC *v.s.* ▲8wC; ∎8wC *v.s.* ▲6wC). Although tissues showed more compaction with a larger deflection compaction from square tissues with the increase of side length, the difference seemed less prominent comparing to the experimental results (Figure 4C). From all the simulation results, the normal strains are more prominent around the standing posts and in the periphery of the tissues, which suggested cell elongation occurred at the tissue boundaries aligned along the geometric sides and exhibited high contractility.

The 6 mm square tissues showed deviation compared to the computational results and experimental observations of the tissues with other shapes. We observed minimal volumetric compaction from the 6 mm square tissues (∎6wC), which deviated from the computational results and the experimental observations of the tissues with other shapes. We hypothesize that due to less mechanical constraints from the shorter distance between the outer posts, tissue inward compaction was compensated by the tissue outward growth, shown as the closing gaps and outward deflections. However, it is difficult to precisely model the tissue growth, which is one of the limitations of the study. In addition, we also hypothesized that active stress fibers within the biological tissues might be one of the missing factors that led to the discrepancies between current computational model and experimental results. We applied one widely used active stress-fiber model (Wang et al., 2013) to our tissue configuration but only observed localized deformation near the post, although our numerical simulations can reproduce reported necking behaviors of a tissue confined by two posts (Wang et al., 2013). In our experimental platform, tissue deformation is under a biaxial loading with a planar configuration and different from the uniaxial loading in the previous model. This might be the reason why our case is dominated by the volumetric contraction effect in comparison to stress-fiber flow effect. However, both factors would play roles in the tissue deformation, whose coupling requires more complicated models and is beyond the scope of the current paper. It will be a critical step for our future study to integrate stress-fiber related plastic model with volumetric active contraction model to seek better alignment between computational and experimental results.

## Conclusion

We have established an *in vitro* mesenchymal tissue model based on hiPSC-MSCs to study the tissue morphological evolution under the mechanical constraints from stiff standing posts. Meanwhile, we also developed a computational model based on volumetric contraction with a time-dependent free-energy function to predict the stress distribution and the corresponding tissue deformation. Comparing to previous works, our study provided more evidence on long-term morphological evolution of large sized biological tissues. Furthermore, our computational model focused on the stress-relaxation behaviors associated with the viscoelastic effect of the tissues, shown as a moduli decay. Our integrated simulations and experiments demonstrated that the ratio of the active contractile modules and the elastic moduli played an important role in determining tissue deformation under constraints.

Our experimental-computational integrated model demonstrated that changing tissue shape and increasing the side length would lead to more prominent tissue remodeling with larger gap extension and deflection compaction. Additionally, the insertion of a center post could alter the mechanical strain distribution and enhance the tissue stability during remodeling processes. According to the current mechanical model, the gap extension was the result of strain concentration on tissue materials around the posts, but these observations cannot be categorized as necking instability as discussed previously.

Compared with the experimental data, the numerical model predicted a larger tissue deflection, which might be attributed to the absence of both plastic deformation of tissues (Nam et al., 2016) and stress-induced anisotropic properties (Bose et al., 2019) in the current computational model. In future study, we will extend the numerical model by incorporating fiber alignment and network reconfiguration that would generate the anisotropic behaviors of biological tissues. Future studies can be explored to analyze and predict the tissue instability and failure under a multi-post configuration, which can provide rational design of functional tissues with reduced local stresses.

## Experimental Section

### Derivation of Mesenchymal Stem Cells From hiPSCs

Differentiation and maintenance of the hiPSC-MSCs have been previously described (Winston et al., 2019). Simply, the hiPSCs were seeded on Geltrex-coated 6-well plates and maintained in Essential 8 (E8) media (Life Technologies, Ca# A1517001). Next, hiPSCs were differentiated into MSCs using a specified differentiation media consisting of 10 ng/ml bFGF (R&D Systems, Ca# 233-FB), 4 μM SB431542 (Stemgent, Ca# 04-0010-10), and 4 μM WNT agonist CHIR99021 (CHIR) (Stemgent, 04-2004) in Essential 6 (E6) media (Life Technologies, A1516401). The differentiated hiPSC-MSCs were maintained in the CTS StemPro MSC SFM media (Life Technologies, A1033201) and passaged every 6 days.

### Fabrication of Tissue Constructs

The micro tissues were generated using a protocol previously established by the lab (Winston et al., 2019). The hiPSC-MSCs were dissociated using trypsin, collected, and pelleted in the high glucose DMEM with 10% FBS and 1% Penicillin-Streptomycin, and then resuspended in collagen I hydrogel (density of 2 mg/ml) prepared based on manufacturer’s instruction (Life Technologies, A1048301). The cell-collagen mixture was loaded into PDMS devices that were cast from ABS molds and formed the tissues within 24 h. The hiPSC-MSC tissues were maintained in culture media (high glucose DMEM, 10% FBS, and 1% pen-strep) for 12 days with media changes every 2 days.

### Fluorescent Microscopy

The tissues were fixed with 4% (vol/vol) paraformaldehyde (PFA) and 0.2% triton for 15 min after washing three times with DPBS. The tissues were incubated in 2% BSA for 30 min for blocking before incubating fluorophore labelled phalloidin (Cytoskeleton Inc., PHDH1) for 2 h at room temperature or overnight at 4°C to visual the F-actin filaments in the cells. DAPI was used to stain the nuclei. Fluorescent images were taken using a Leica upright fluorescent microscope with Thunder deconvolution processing.

### Scanning Electron Microscopy

The tissue was fixed with 4% (vol/vol) PFA for 15 min and refrigerated at 4°C overnight. The tissue was then washed once in 15, 30, 50, 70 and 95% ethanol for 15 min each time and finally with 100% ethanol three times to dehydrate. The tissue was then left in the vacuum oven overnight to dry. Sections of the tissue were fixed and mounted on stubs before being coated with 10 nm gold/palladium layer. Samples were viewed and imaged under scanning electron microscope JEOL JSM-IT100.

### Gap and Deflection Measurement

Tissue morphological changes were imaged and examined using a Nikon Eclipse Ti microscope with the Zyla 4.2 PLUS sCMOS camera at ×4 magnification and NIS Element software. As a result of large tissue size, nine segments of the construct were imaged and then stitched together to form a complete image. The gap and deflection at each side of the tissue were measured using ImageJ and averaged for one tissue construct. To measure each gap and deflection of a triangular tissue, an equilateral triangle was first created by linking the center of three outer posts. On the reference line between one vertex and the geometric center of the equilateral triangle, the gap was measured between the edge of the tissue and the corresponding vertex. On the reference line perpendicular to the side from the geometric center of the equilateral triangle, the deflection was measured between the edge of the tissue and the geometric center. To measure each gap and deflection of a square tissue, a square was first created by linking the center of four outer posts. On the reference line between one vertex and the geometric center of the square, the gap was measured between the edge of the tissue and the corresponding vertex. On the reference line perpendicular to the side from the geometric center of the square, the deflection was measured between the edge of the tissue and the geometric center (Supplementary Figure S2). All measurements were normalized to Day 1.

### Statistical Analysis

All the conditions had at least four tissue samples for statistical analysis. Data was plotted as line or box plots with all the data points included. For the comparison between two individual groups, a two-sided Student’s t-test was used, and *p* ≤ 0.05 was considered significant. For the comparison between more than two groups, one-way analysis of variance (ANOVA) with Tukey’s multiple comparisons test was performed to determine significance between groups, and *p* ≤ 0.05 was considered significant.

### Computational Modeling

The computational modeling was implemented through a finite element software ABAQUS/Explicit. A VUMAT user material subroutine was developed to simulate the contraction of tissue material. The tissue formed a hard and frictionless contact with the stiff pillars that were modeled as rigid cylinders. The interface displacement of the tissue on the pillar surface was fixed to prevent the detachment of tissue from the center pillars during the contraction. An 8-node linear hexahedral element was used with reduced integration (C3D8R) and 0.2 mm mesh size. To mimic the quasi-static process, the mass scaling was used to keep the kinetic energy negligible compared to the deformation energy. The geometric parameters were identical to the experimental settings. The material parameters used here included: the contraction modulus *η* = 11 kPa, the Young’s modulus *E*_0_ = 18.33 kPa, and the Poisson’s ratio *υ* = 0.3 (Mailand et al., 2019). The initial shear and bulk moduli were calculated as: *μ*_0_ = *E*_0_/(2 (1+*υ*)), *κ*_0_ = *E*_0_/(3 (1–2*υ*)). The Day 1 tissues were modeled corresponding to the contraction of the initial elastic moduli. The viscoelastic relaxation started in the following days with a characteristic relaxation time *τ* of 4 days. The materials parameters are used for all the simulations of triangle and square tissues with different designs of post configurations.

## Data Availability

The original contributions presented in the study are included in the article/Supplementary Material, further inquiries can be directed to the corresponding authors.

## Abbreviations

3D, three-dimensional; hiPSC-MSCs, mesenchymal stromal cells derived from human induced pluripotent stem cells; SEM, scanning electron microscope; ▲6woC, triangular tissue with side length of 6 mm without a center post; ▲6wC, triangular tissue with side length of 6 mm with a center post; ▲8woC, triangular tissue with side length of 8 mm without a center post; ▲8wC, triangular tissue with side length of 8 mm with a center post; ▲8wC-R, triangular tissue with side length of 8 mm with a small center post; ∎6wC, square tissue with side length of 6 mm with a center post; ∎8wC, square tissue with side length of 8 mm with a center post.

## References

[B1] Bose P. Eyckmans J. Nguyen T. D. Chen C. S. Reich D. H. (2019). Effects of Geometry on the Mechanics and Alignment of Three-Dimensional Engineered Microtissues. ACS Biomater. Sci. Eng. 5, 3843–3855. 10.1021/acsbiomaterials.8b01183 33438424

[B2] Christensen R. K. von Halling Laier C. Kiziltay A. Wilson S. Larsen N. B. (2020). 3D Printed Hydrogel Multiassay Platforms for Robust Generation of Engineered Contractile Tissues. Biomacromolecules 21, 356–365. 10.1021/acs.biomac.9b01274 31860278

[B3] Deshpande V. S. McMeeking R. M. Evans A. G. (2006). A Bio-Chemo-Mechanical Model for Cell Contractility. Proc. Natl. Acad. Sci. 103, 14015–14020. 10.1073/pnas.0605837103 16959880 PMC1560932

[B4] Dey K. Roca E. Ramorino G. Sartore L. (2020). Progress in the Mechanical Modulation of Cell Functions in Tissue Engineering. Biomater. Sci. 8, 7033–7081. 10.1039/d0bm01255f 33150878

[B5] Dubois S. J. Kalashnikov N. Moraes C. (2019). Robust and Precise Wounding and Analysis of Engineered Contractile Tissues. Tissue Eng. C: Methods 25, 677–686. 10.1089/ten.tec.2019.0123 31411125

[B6] Gibbons G. H. Dzau V. J. (1994). The Emerging Concept of Vascular Remodeling. N. Engl. J. Med. 330, 1431–1438. 10.1056/NEJM199405193302008 8159199

[B7] Hinz B. Phan S. H. Thannickal V. J. Prunotto M. Desmoulière A. Varga J. (2012). Recent Developments in Myofibroblast Biology. Am. J. Pathol. 180, 1340–1355. 10.1016/j.ajpath.2012.02.004 22387320 PMC3640252

[B8] Kim J. Mailand E. Ang I. Sakar M. S. Bouklas N. (2020). A Model for 3D Deformation and Reconstruction of Contractile Microtissues. Soft Matter 17, 10198. 10.1039/d0sm01182g 33118554

[B9] Legant W. R. Chen C. S. Vogel V. (2012). Force-induced Fibronectin Assembly and Matrix Remodeling in a 3D Microtissue Model of Tissue Morphogenesis. Integr. Biol. 4, 1164–1174. 10.1039/c2ib20059g PMC3586566 22961409

[B10] Legant W. R. Pathak A. Yang M. T. Deshpande V. S. McMeeking R. M. Chen C. S. (2009). Microfabricated Tissue Gauges to Measure and Manipulate Forces from 3D Microtissues. Proc. Natl. Acad. Sci. 106, 10097–10102. 10.1073/pnas.0900174106 19541627 PMC2700905

[B11] Liu A. S. Wang H. Copeland C. R. Chen C. S. Shenoy V. B. Reich D. H. (2016). Matrix Viscoplasticity and its Shielding by Active Mechanics in Microtissue Models: Experiments and Mathematical Modeling. Sci. Rep. 6, 33919. 10.1038/srep33919 27671239 PMC5037370

[B12] Mailand E. Li B. Eyckmans J. Bouklas N. Sakar M. S. (2019). Surface and Bulk Stresses Drive Morphological Changes in Fibrous Microtissues. Biophysical J. 117, 975–986. 10.1016/j.bpj.2019.07.041 PMC6731460 31427068

[B13] Malandrino A. Trepat X. Kamm R. D. Mak M. (2019). Dynamic Filopodial Forces Induce Accumulation, Damage, and Plastic Remodeling of 3D Extracellular Matrices. Plos Comput. Biol. 15, e1006684. 10.1371/journal.pcbi.1006684 30958816 PMC6472805

[B14] Nam S. Lee J. Brownfield D. G. Chaudhuri O. (2016). Viscoplasticity Enables Mechanical Remodeling of Matrix by Cells. Biophysical J. 111, 2296–2308. 10.1016/j.bpj.2016.10.002 PMC5113260 27851951

[B15] Nour S. Baheiraei N. Imani R. Khodaei M. Alizadeh A. Rabiee N. (2019). A Review of Accelerated Wound Healing Approaches: Biomaterial- Assisted Tissue Remodeling. J. Mater. Sci. Mater. Med. 30, 120. 10.1007/s10856-019-6319-6 31630272

[B16] Pathak A. Deshpande V. S. McMeeking R. M. Evans A. G. (2008). The Simulation of Stress Fibre and Focal Adhesion Development in Cells on Patterned Substrates. J. R. Soc. Interf. 5, 507–524. 10.1098/rsif.2007.1182 PMC2375958 17939980

[B17] Rajagopal V. Holmes W. R. Lee P. V. S. (2018). Computational Modeling of Single-Cell Mechanics and Cytoskeletal Mechanobiology. Wiley Interdiscip. Rev. Syst. Biol. Med. 10, e1407. 10.1002/wsbm.1407 PMC5836888 29195023

[B18] Ramade A. Legant W. R. Picart C. Chen C. S. Boudou T. (2014). Microfabrication of a Platform to Measure and Manipulate the Mechanics of Engineered Microtissues. Methods Cel Biol 121, 191–211. 10.1016/b978-0-12-800281-0.00013-0 24560511

[B19] Rodriguez-Porcel M. Zhu X.-Y. Chade A. R. Amores-Arriaga B. Caplice N. M. Ritman E. L. (2006). Functional and Structural Remodeling of the Myocardial Microvasculature in Early Experimental Hypertension. Am. J. Physiology-Heart Circulatory Physiol. 290, H978–H984. 10.1152/ajpheart.00538.2005 PMC1363360 16214846

[B20] Rouwkema J. Khademhosseini A. (2016). Vascularization and Angiogenesis in Tissue Engineering: Beyond Creating Static Networks. Trends Biotechnol. 34, 733–745. 10.1016/j.tibtech.2016.03.002 27032730

[B21] Sakar M. S. Eyckmans J. Pieters R. Eberli D. Nelson B. J. Chen C. S. (2016). Cellular Forces and Matrix Assembly Coordinate Fibrous Tissue Repair. Nat. Commun. 7, 11036. 10.1038/ncomms11036 26980715 PMC4799373

[B22] Smith P. C. Martínez C. Martínez J. McCulloch C. A. (2019). Role of Fibroblast Populations in Periodontal Wound Healing and Tissue Remodeling. Front. Physiol. 10, 270. 10.3389/fphys.2019.00270 31068825 PMC6491628

[B23] Song W. Chiu A. Wang L.-H. Schwartz R. E. Li B. Bouklas N. (2019). Engineering Transferrable Microvascular Meshes for Subcutaneous Islet Transplantation. Nat. Commun. 10, 4602. 10.1038/s41467-019-12373-5 31601796 PMC6787187

[B24] Walker M. Godin M. Harden J. L. Pelling A. E. (2020). Time Dependent Stress Relaxation and Recovery in Mechanically Strained 3D Microtissues. APL Bioeng. 4, 036107. 10.1063/5.0002898 32984751 PMC7500532

[B25] Wang H. Svoronos A. A. Boudou T. Sakar M. S. Schell J. Y. Morgan J. R. (2013). Necking and Failure of Constrained 3D Microtissues Induced by Cellular Tension. Proc. Natl. Acad. Sci. 110, 20923–20928. 10.1073/pnas.1313662110 24324149 PMC3876233

[B26] Winston T. S. Suddhapas K. Wang C. Ramos R. Soman P. Ma Z. (2019). Serum-Free Manufacturing of Mesenchymal Stem Cell Tissue Rings Using Human-Induced Pluripotent Stem Cells. Stem Cell Int 2019, 5654324. 10.1155/2019/5654324 PMC6350554 30766604

[B27] Zhao R. Boudou T. Wang W.-G. Chen C. S. Reich D. H. (2013). Decoupling Cell and Matrix Mechanics in Engineered Microtissues Using Magnetically Actuated Microcantilevers. Adv. Mater. 25, 1699–1705. 10.1002/adma.201203585 23355085 PMC4037409

