# Supplementary material for: Controlling Mesenchyme Tissue Remodeling via Spatial Arrangement of Mechanical Constraints
Source: Front Bioeng Biotechnol. 2022 Feb 18;10:833595. doi: 10.3389/fbioe.2022.833595 (PMC8896258; doi:10.3389/fbioe.2022.833595)
Supplement: Supplementary file 1 [file DataSheet1.PDF]

SUPPLEMENTAL MATERIALS

Controlling Mesenchyme Tissue Remodeling via Spatial Arrangement of Mechanical Constraint

Tackla S. Winston[1,2†], Chao Chen[2,3†], Kantaphon Suddhapas[1,2$,] Bearett A. Tarris[1,2], Saif Elattar[4], Shiyang Sun[1,2], Teng Zhang[2,3], Zhen Ma[1,2*]*

[1]Department of Biomedical & Chemical Engineering, Syracuse University, Syracuse, NY 13244
[2]BioInspired Syracuse Institute for Materials and Living Systems, Syracuse University, Syracuse, NY 13244
[3]Department of Mechanical & Aerospace Engineering, Syracuse University, Syracuse, NY 13244
[4]Department of Chemical & Petroleum Engineering, University of Kansas, Lawrence, KS 66045

[$]Current Address: Department of Bioengineering, University of Illinois Urbana Champaign, Urbana, IL 61801

[†]These two authors contributed to this work equally.

[*]Corresponding Authors:
Teng Zhang
Department of Mechanical & Aerospace Engineering
BioInspired Syracuse Institute for Materials and Living Systems
Syracuse University, Syracuse, NY 13244
ORCID: 0000-0001-5001-8485
Email: tzhang48@syr.edu

Zhen Ma
Department of Biomedical & Chemical Engineering
BioInspired Syracuse Institute for Materials and Living Systems
Syracuse University, Syracuse, NY 13244
ORCID: 0000-0001-5228-105X
Email: zma112@syr.edu

Supplemental Figures

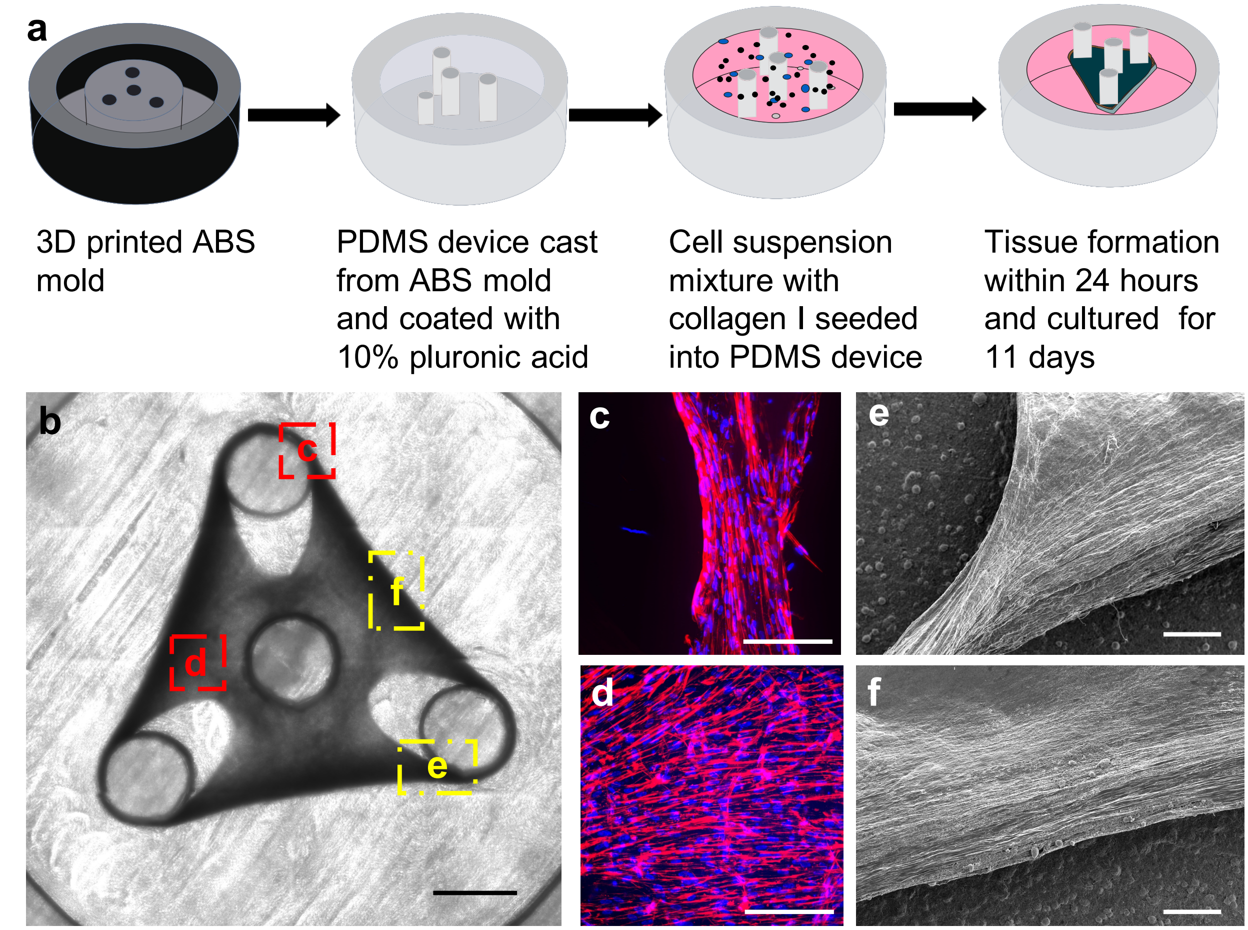

Figure S1: 3D mesenchymal tissue fabrication with multi-post designs. (a) schematic representation of 3D tissue fabrication of hiPSC-MSCs that were suspended in collagen scaffold and seeded into the PDMS devices with stiff cylindrical standing posts. (b) A representative image of a triangular mesenchymal tissue with center post (Scale Bar = 2 mm). Dash boxes indicated the locations for the fluorescent images c & d (red) and the SEM images e & f. Actin staining showed (c) tissue thinning and fiber elongation around the post and (d) fiber orientation within the tissue body. SEM images showed similar fiber elongation (e) around the post and (f) at the tissue edge.

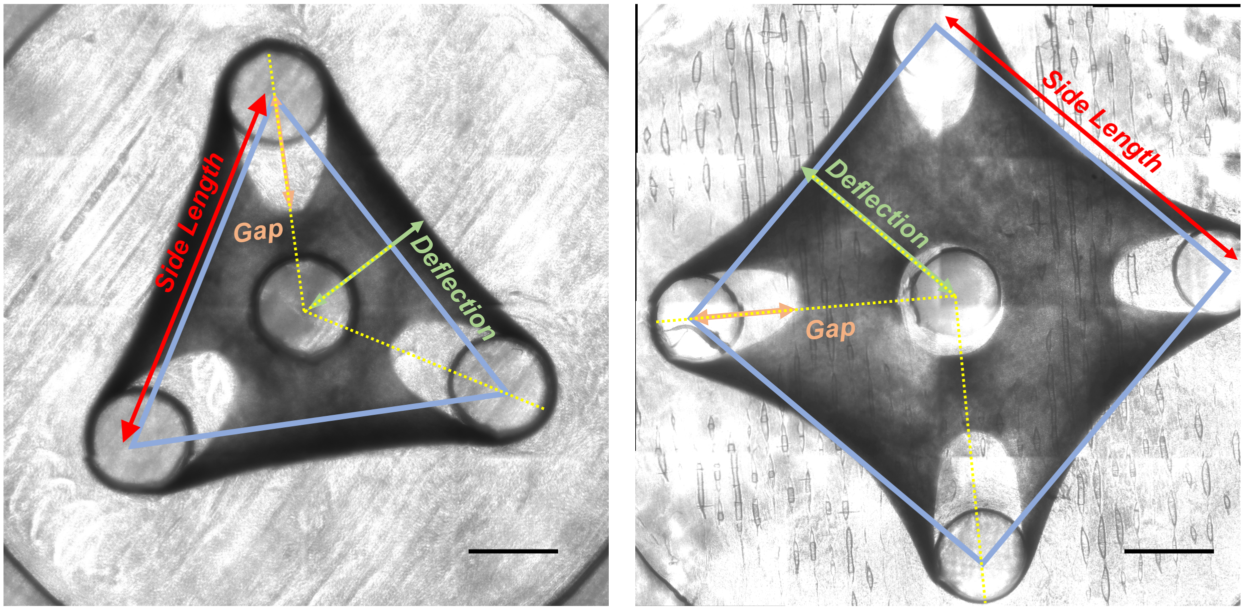

Figure S2: Measurements of tissue morphological evolution for triangular and square tissues. The side length was defined as the distance between the center of two outer posts (*red arrow lines*). By linking the center of outer posts, an arbitrary equilateral triangle can be created for a triangular tissue, and an arbitrary square can be created for a square tissue (*blue lines*). On the reference lines (*yellow dash lines*) between one vortex and the geometric center of arbitrary equilateral triangle or square, the gap can be measured between the inner tissue edge and the vortex (*pink arrow lines*). On the reference lines (*yellow dash lines*) perpendicular to the side from the geometric center of the triangle or the square, the deflection can be measured between the outer tissue edge and the geometric center (*green arrow lines*). Scale Bar = 2 mm.

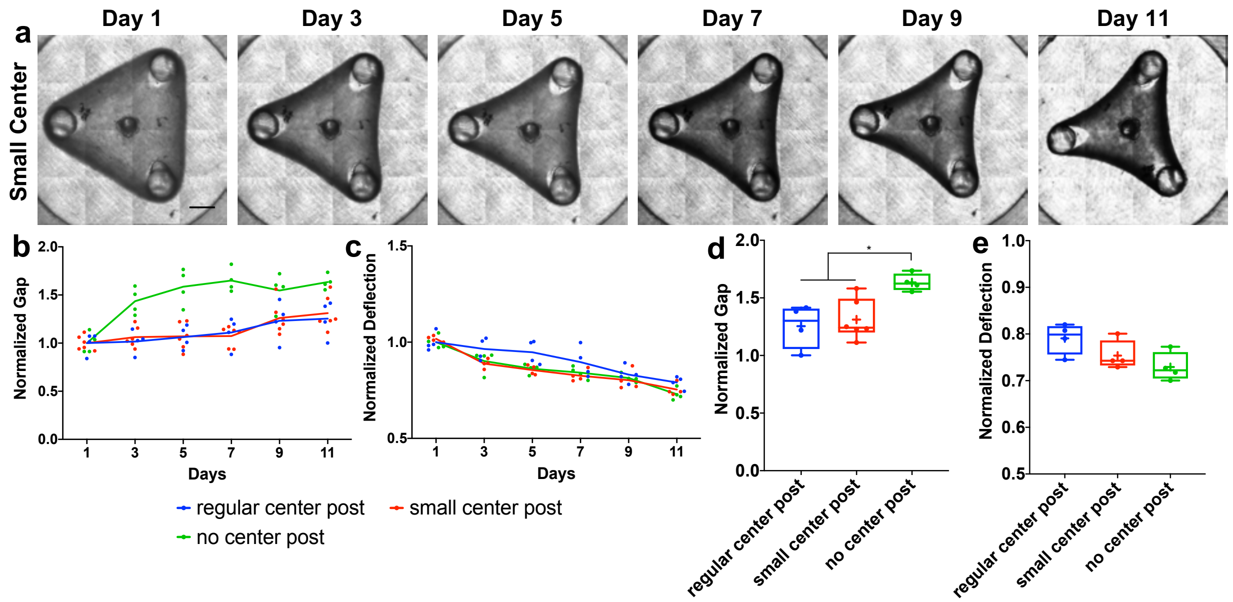

Figure S3: Impact of the size of the center post on tissue remodeling. (a) Representative images of tissue remodeling over 11 days for the triangular tissues with small center posts. (b) Tissue thinning around the post led to gap extension, and (c) inward remodeling at the edge led to deflection compaction. The gap and deflection were measured every two days for tissue comparison (▲8woC, ▲8wC and ▲8wC-R). Taking the measurements on Day 11, (d) the gap of ▲8woC tissues was significantly larger than the tissues with a center post (▲8wC and ▲8wC-R), and (e) the deflection among these tissues was not significantly different. Scale bar: 1mm. *$p < 0.05$.

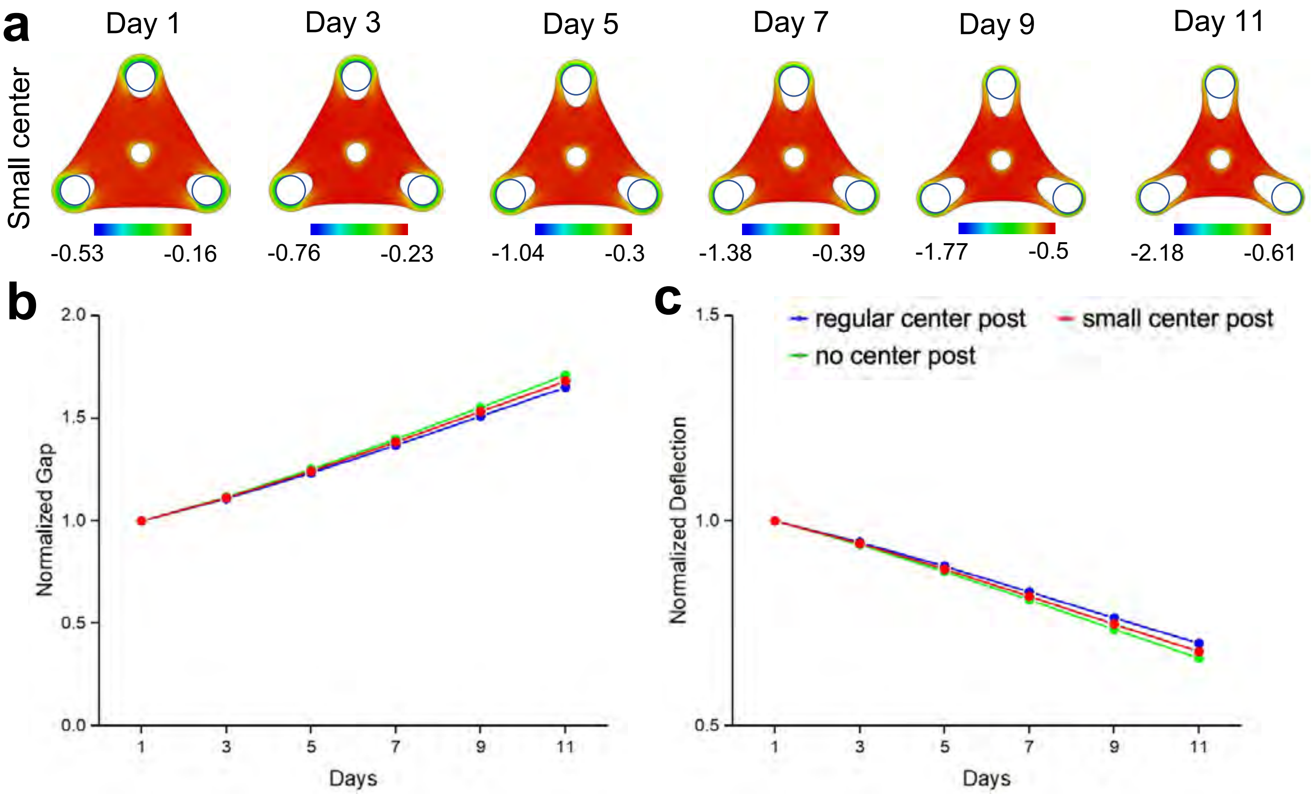

Figure S4: Simulation results of morphogenic evolution of triangular tissues with small center post. a) A volumetric contraction model with a time-dependent free-energy function was used to simulate the morphological evolution of the triangular tissues. The color represents the minimal principal logarithmic strain distribution. The computational model was able to replicate the trends in morphological changes of gap extension and deflection compaction that were observed from the experimental model. For the tissue comparison (▲8woC, ▲8wC, and ▲8wC-R), there are small differences on (b) gap and (c) deflection measurements over 11 days among these tissues with different center post designs.
